# Supplementary material for: 326K at E Protein Is Critical for Mammalian Adaption of TMUV
Source: Viruses. 2023 Dec 1;15(12):2376. doi: 10.3390/v15122376 (PMC10747068; doi:10.3390/v15122376)
Supplement: Supplementary file 1 [file viruses-15-02376-s001.zip › viruses-2691414-supplementary.pdf]

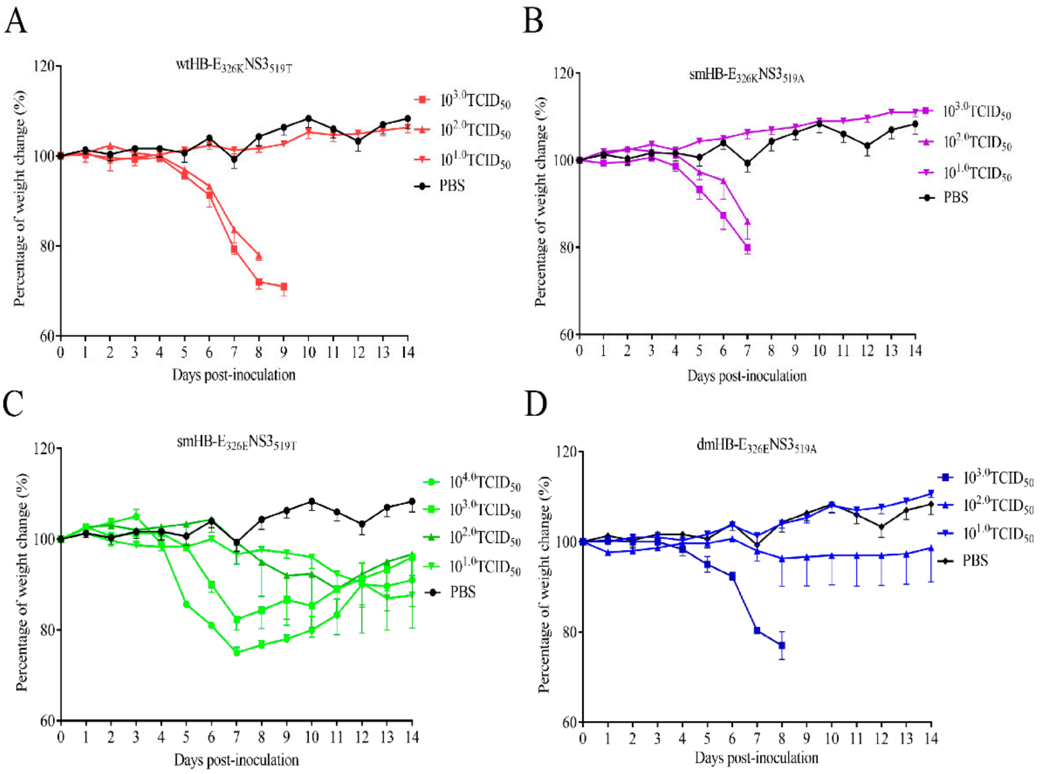


Figure S1. Weight change curves of mice. (A to D) Five-week-old BALB/c mice were infected by the i.c. route with four viruses at different doses (10^1.0, 10^2.0, 10^3.0 or 10^4.0 $TCID_{50}$), respectively or mock infected. Animals were monitored daily for the appearance of symptoms and weight changes were recorded during the two weeks period of observation.

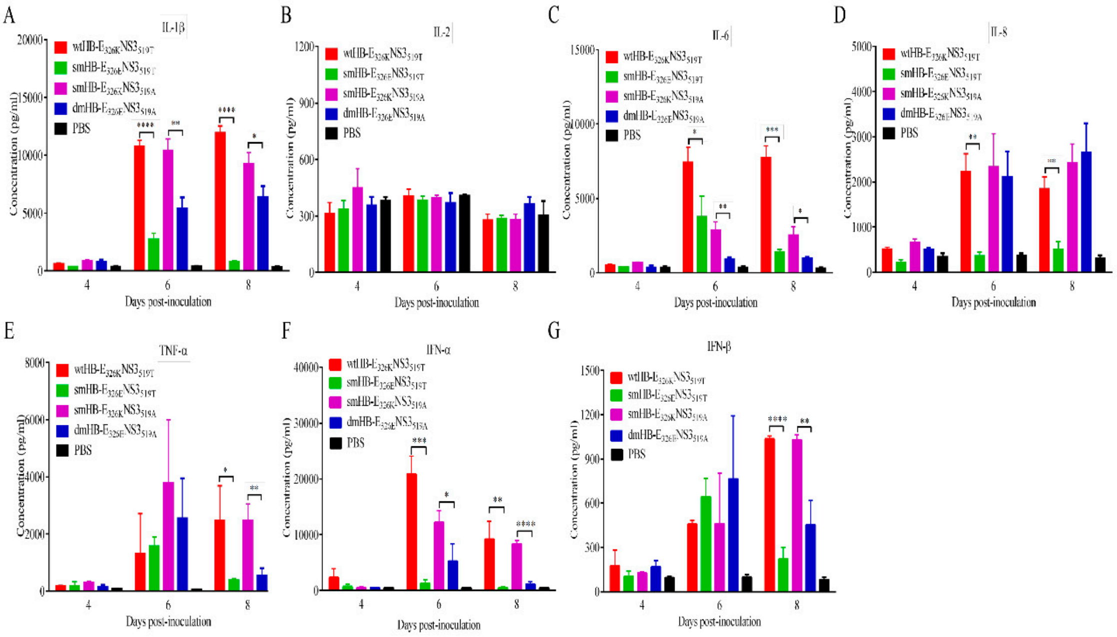


Figure S2. The wtHB-$E_{326K}NS3_{519T}$ and mutant viruses induce different protein levels inflammatory cytokines in the mouse brain. Five-week-old BALB/c mice were infected by the i.c. route with virus at a dose 10^3.0 $TCID_{50}$. Supernatants of infected mouse brain homogenates

were collected for measurement of pro-inflammatory cytokines(A-E) and IFN-α/β (F and G) at protein levels by ELISA. (****, $p < 0.0001$; ***, $p < 0.001$; **, $p < 0.01$; *, $p < 0.05$).

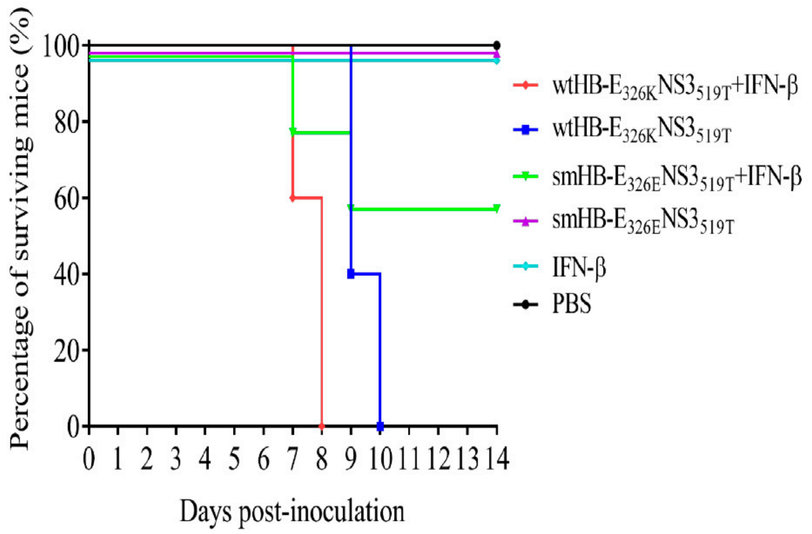


Figure S3. The death of infected mice was promoted by i.c. injection of IFN-β. Five-week-old BALB/c mice were infected by the i.c. route with virus at a dose 10^3.0 $TCID_{50}$. Human recombinant IFN-β was injected into the brain of mice at a dose of 3000 U at 3 dpi and 5 dpi to observe the survival of the mice.

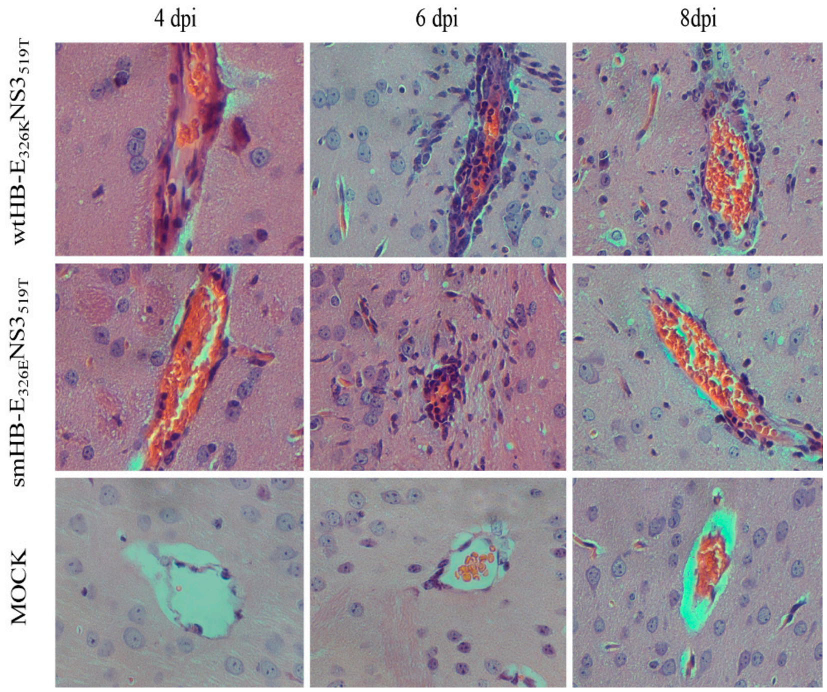


Figure S4. Histopathologic analysis of the hematoxylin and eosin (HE)-stained brain tissue of mice infected. Five-week-old female BALB/c mice were infected by the i.c. route with wtHB-$E_{326K}NS3_{519T}$ and smHB-$E_{326E}NS3_{519T}$ at a dose 10^3.0 $TCID_{50}$. Brain tissues of infected mice was collected at 4, 6, and 8 dpi for histopathological analysis.

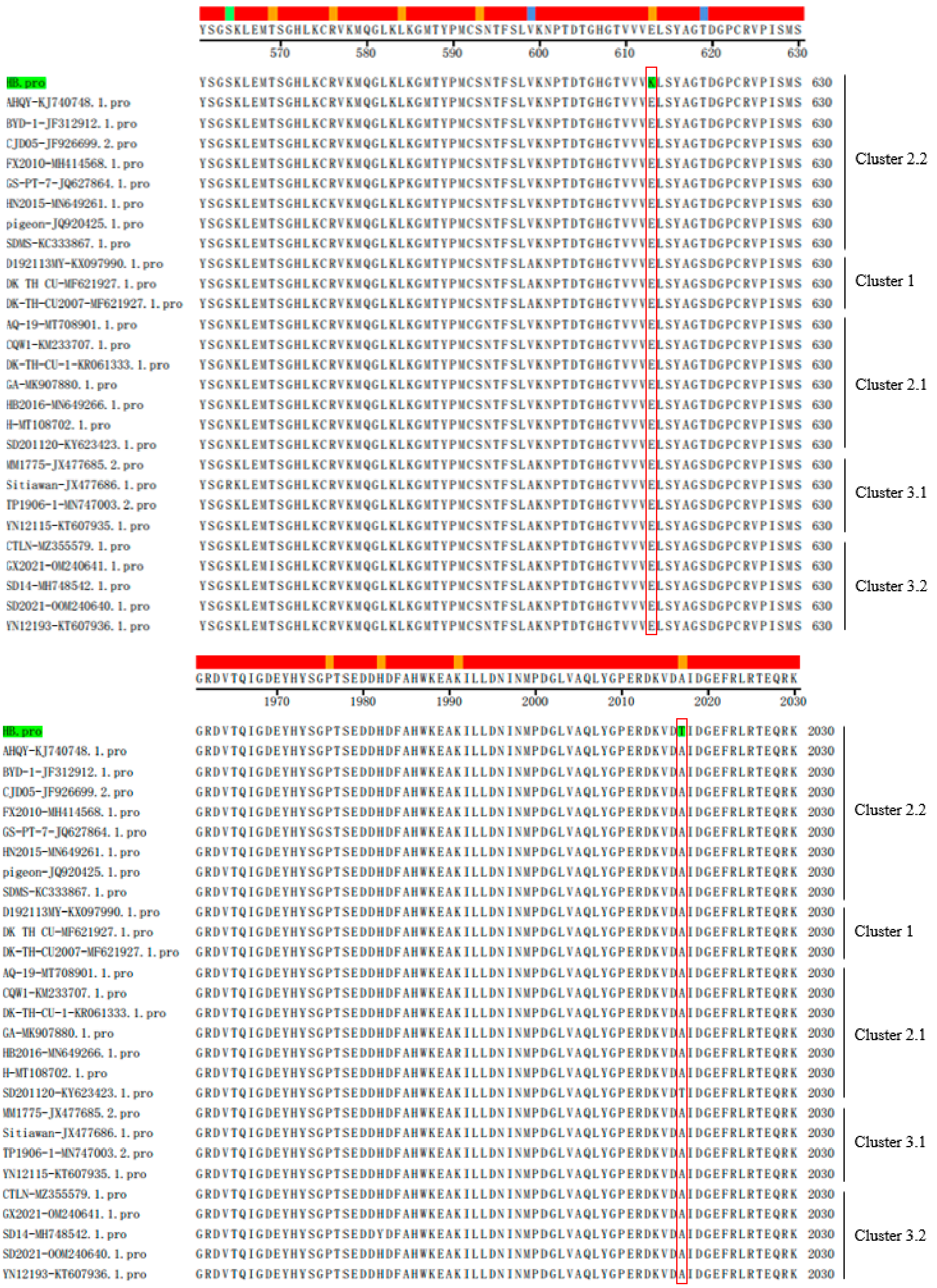

Figure S5. Sequence analysis results. The sequencing results were analyzed using DNASTAR software. The reference virus strain information have been supplemented in Table S2.

Table S1. Primers used in the study

| Primer name | Sequence (5′to 3') | Positions | Application |
|---|---|---|---|
| HBRT1 | ACACATCGGGTAGGTC | 1856-1871 | Specific reverse transcription primer |
| HBRT2 | CCTTCTCAAAATCTCC | 3578-3593 | Specific reverse transcription primer |
| HBRT3 | ATCTCTGTGATGCTCC | 4404-4419 | Specific reverse transcription primer |
| HBRT4 | GTCAACCTTGTCCCGC | 2433-2457 | Specific reverse transcription primer |
| HBRT5 | TGCCGTCGCTGGTCTCAA | 3806-3831 | Specific reverse transcription primer |
| HBRT6 | TAACCTCTCACTTCCT | 3656-3680 | Specific reverse transcription primer |
| HBRT7 | AGACTCTGTGTTCTAC | 10957-10991 | Specific reverse transcription primer |
| HBT7-1F | CCCGGGTAATACGACTCACTATAGGGA GAAGTTCATCTGTGTGAACTTATTCC | 1-27 | Amplifying HB |
| HB-956R | GCTGTACGCTGGGGC | 942-956 | Amplifying HB |
| HB-942F | GCCCCAGCGTACAGCTT | 942-958 | Amplifying HB |
| HB-2459R | GGCATTGACATTTACTGCC | 2441-2459 | Amplifying HB |
| HB-2433F | GTCTTCCTGGCAGTAAATGTCAATG | 2433-2457 | Amplifying HB |
| HB-3831R | ATCTGCTTTCCTGATTGCTCCACTTC | 3806-3831 | Amplifying HB |
| HB-3656F | AGGAATCACGTACAGTGATCTGGTC | 3656-3680 | Amplifying HB |
| HB-10991R | AGACTCTGTGTTCTACCACCACCAGCC ACACTTTC | 10957-10991 | Amplifying HB |
| E-K326E-F | GGCACTGTCGTGGTG**GAA**TTGT | 1917-1938 | E K326E mutation |
| E-K326E-R | ACAA**TTC**CACCACGACAGTGCC | 1917-1938 | E K326E mutation |
| NS3-T519A-F | GACAAGGTTGAC**GCA**ATTGATG | 6132-6153 | NS3 T519A mutation |
| NS3-T519A-R | CATCAAT**TGC**GTCAACCTTGTC | 6132-6153 | NS3 T519A mutation |
| HB-E-1936-F | TGTCTTATGCAGGTACCGATG | 1936-2040 | qRT-PCR primer |
| HB-E-2040-R | CGTATGGGTTGACTGTTATCA | 1936-2040 | qRT-PCR primer |
| HB-E-probe | FAM-AGTTCCCATATCCATGTC-TAMRA | 1967-1984 | Probe primer |
| qMouse-IL-1β-F | GGTGTGTGACGTTCCCATTA | _ | qRT-PCR primer |
| qMouse-IL-1β-R | ATTGAGGTGGAGAGCTTTCAG | _ | qRT-PCR primer |
| qMouse-IL-2-F | TGAGCAGGATGGAGAATTACAG | _ | qRT-PCR primer |
| qMouse-IL-2-R | GAGGTCCAAGTTCATCTTCTAGG | _ | qRT-PCR primer |
| qMouse-IL-6-F | CTTCCATCCAGTTGCCTTCT | _ | qRT-PCR primer |

| | | | |
|---|---|---|---|
| qMouse-IL-6-R | CTCCGACTTGTGAAGTGGTATAG | _ | qRT-PCR primer |
| qMouse-IL-8-F | TGGGAGAATTCAAGGTGGATAAG | _ | qRT-PCR primer |
| qMouse-IL-8-R | GACAGCATCTGGCAGAATAGAG | _ | qRT-PCR primer |
| qMouse-TNF-α-F | GTTCTATGGCCCAGACCCTCA | _ | qRT-PCR primer |
| qMouse-TNF-α-R | CAGCAAGCATCTATGCACTTAG | _ | qRT-PCR primer |
| qMouse-IFN-α-F | CCCGCAGGAGAAGGTGGAT | _ | qRT-PCR primer |
| qMouse-IFN-α-R | GAGCTGCTGGTGGAGGTCA | _ | qRT-PCR primer |
| qMouse-IFN-β-F | GCTGCGTTCCTGCTGTGCT | _ | qRT-PCR primer |
| qMouse-IFN-β-R | CATCTTCTCCGTCATCTCCA | _ | qRT-PCR primer |
| qMouse-IFN-γ-F | ACTGGCAAAAGGATGGTGAC | _ | qRT-PCR primer |
| qMouse-IFN-γ-R | TGAGCTCATTGAATGCTTGG | _ | qRT-PCR primer |
| qMouseGAPDH-F | GAGGCCGGTGCTGAGTATGT | _ | qRT-PCR primer |
| qMouseGAPDH-R | CGGCAGAAGGGGCGGAGATG | _ | qRT-PCR primer |
| qMouse-RIG-I-F | GAGCCAGCGGAGATAACAATA | _ | qRT-PCR primer |
| qMouse-RIG-I-R | CCCACGTACTCATAGAGAATGAC | _ | qRT-PCR primer |
| qMouse-MDA5-F | GCTAAAGACGGAAATCGCAAAG | _ | qRT-PCR primer |
| qMouse-MDA5-R | GAATGTCTCCAGGTGGCTATATG | _ | qRT-PCR primer |
| qMouse-TLR3-F | ACCTCCAGAAGAACCTCATAAC | _ | qRT-PCR primer |
| qMouse-TLR3-R | GAACGGATTGAAGCGCATATC | _ | qRT-PCR primer |
| qMouse-TLR7-F | AACCTTTCCCAGAGCATACAG | _ | qRT-PCR primer |
| qMouse-TLR7-R | GGAGCCTCTGATGAGACAAATAA | _ | qRT-PCR primer |
| qMouse-IRF3-F | GTCTTAAGGAGCTGTTAGAGATGG | _ | qRT-PCR primer |
| qMouse-IRF3-R | TGGTCAGAGGTAAGGGAGATAG | _ | qRT-PCR primer |
| qMouse-IRF7-F | TTCTGCAGTACAGCCACATAC | _ | qRT-PCR primer |
| qMouse-IRF7-R | GCATAGGGTTCCTCGTAAACA | _ | qRT-PCR primer |

Table S2. The reference virus strain information

| Strains | GenBank No. | Source | Date |
|---|---|---|---|
| FX2010 | MH414568.1 | Duck | 2010 |
| BYD-1 | JF312912.1 | Duck | 2010 |
| pigeon | JQ920425.1 | pigeon | 2012 |
| CJD05 | JF926699.2 | Chicken | 2010 |
| AHQY | KJ740748.1 | Duck | 2013 |
| HN2015 | MN649261.1 | Duck | 2015 |
| SDMS | KC333867.1 | Mosquito | 2012 |
| GS-PT-7 | JQ627864.1 | Goose | 2010 |
| CQW1 | KM233707.1 | Duck | 2013 |
| SD201120 | KY623423.1 | Duck | 2011 |
| GA | MK907880.1 | Duck | 2018 |
| AQ-19 | MT708901.1 | Goose | 2019 |
| HB2016 | MN649266.1 | Duck | 2016 |
| H | MT108702.1 | Duck | 2019 |
| DK/TH/CU-1 | KR061333.1 | Duck | 2013 |
| D1921/1/3MY | KX097990.1 | Duck | 2012 |
| D1977/1/MY | KX097989.1 | Duck | 2012 |
| DK-TH-CU2007 | MF621927.1 | Duck | 2007 |
| Sitiawan | JX477686.1 | Chicken | 2002 |
| MM1775 | JX477685.2 | Mosquito | 1995 |
| TP1906 | MN747003.2 | Mosquito | 2019 |
| YN12115 | KT607935.1 | Mosquito | 2012 |
| GX2021 | OM240641.1 | Chicken | 2021 |
| SD2021 | OM240640.1 | Chicken | 2021 |
| YN12193 | KT607936.1 | Mosquito | 2012 |
| SD14 | MH748542.1 | Duck | 2014 |
| CTLN | MZ355579.1 | Chicken | 2020 |
